# Effectiveness of generative AI-large language models’ recognition of veteran suicide risk: a comparison with human mental health providers using a risk stratification model

**DOI:** 10.3389/fpsyt.2025.1544951

**Published:** 2025-04-03

**Authors:** Sean A. Lauderdale, Randee Schmitt, Breanna Wuckovich, Natashaa Dalal, Hela Desai, Shealyn Tomlinson

**Affiliations:** Department of Psychological and Behavioral Sciences, University of Houston – Clear Lake, Houston, TX, United States

**Keywords:** veterans, suicide, risks, artificial intelligence, risk stratification model

## Abstract

**Background:**

With over 6,300 United States military veterans dying by suicide annually, the Veterans Health Administration (VHA) is exploring innovative strategies, including artificial intelligence (AI), for suicide risk assessment. Machine learning has been predominantly utilized, but the application of generative AI-large language models (GAI-LLMs) remains unexplored.

**Objective:**

This study evaluates the effectiveness of GAI-LLMs, specifically ChatGPT-3.5, ChatGPT-4o, and Google Gemini, in using the VHA’s Risk Stratification Table for identifying suicide risks and making treatment recommendations in response to standardized veteran vignettes.

**Methods:**

We compared the GAI-LLMs’ assessments and recommendations for both acute and chronic suicide risks to evaluations by mental health care providers (MHCPs). Four vignettes, representing varying levels of suicide risk, were used.

**Results:**

GAI-LLMs’ assessments showed discrepancies with MHCPs, particularly rating the most acute case as less acute and the least acute case as more acute. For chronic risk, GAI-LLMs’ evaluations were generally in line with MHCPs, except for one vignette rated with higher chronic risk by the GAI-LLM. Variation across GAI-LLMs was also observed. Notably, ChatGPT-3.5 showed lower acute risk ratings compared to ChatGPT-4o and Google Gemini, while ChatGPT-4o identified higher chronic risk ratings and recommended hospitalization for all veterans. Treatment planning by GAI-LLMs was predicted by chronic but not acute risk ratings.

**Conclusion:**

While GAI-LLMs offers potential suicide risk assessment comparable to MHCPs, significant variation exists across different GAI-LLMs in both risk evaluation and treatment recommendations. Continued MHCP oversight is essential to ensure accuracy and appropriate care.

**Implications:**

These findings highlight the need for further research into optimizing GAI-LLMs for consistent and reliable use in clinical settings, ensuring they complement rather than replace human expertise.

## Introduction

United States veterans’ suicide rates remain substantial and represent a major cause of preventable morbidity (1). Historically, veterans have faced a disproportionately higher risk of suicide compared to the general population (2). According to the 2023 National Veteran Suicide Prevention Report (1), there were 6,392 veteran deaths by suicide in 2021, and suicide mortality was the second-leading cause of death among veterans under the age of 45 years. Men and women veterans have higher rates of suicide by firearms compared to non-veterans, with the risk of women veteran suicide rate by firearm greatly exceeding (281.1%) those of non-veteran women. Early detection is crucial in suicide prevention (3, 4) and evidence from investigations incorporating screening, education, and active risk monitoring have produced reductions in suicide attempts across active duty military members and veterans (5). Despite these efforts and the substantial resource investment by the Veterans Health Administration (VHA; 6), veterans’ suicide rates continue an upward trajectory (1), indicating a need to develop innovative strategies for veteran suicide assessment and prevention (5).

In response, the VHA has implemented multiple strategies to detect, screen, and prevent veteran suicide. One strategy is the use of artificial intelligence (AI). At present, the VHA has relied on a single AI strategy, known as machine learning (ML; 7), to detect risk of veteran suicide risk. Machine learning employs statistical algorithms to analyze data and flag critical risks found in records, such as electronic health records (8). Introduced in 2017, the Recovery and Engagement and Coordination for Health-Veteran Enhanced Treatment (REACH VET; 9, 10) algorithm scans veterans’ electronic health records for 61 pre-identified risk factors that place veterans in the top.1% risk for suicide. Once identified, veterans are contacted by their most recent provider for intervention. An evaluation of REACH VET that included veterans before and after implementation indicated that REACH VET use produced substantial reductions in suicide attempts and mental health admissions as well as increased completed outpatient appointments and suicide safety plans. Unfortunately, suicide mortality rates did not change according to the evaluation, but this may have been due to low statistical power (10). Numerous other investigations have also found that ML can be an effective strategy to detect suicide risks in community samples (11). Although the REACH VET outcomes are promising, substantial concerns still exist, including latency of risk identification (up to two weeks; 9) and use of risks largely predictive of suicide in White, male veterans, excluding suicide risks of other veteran populations (e.g., women, minorities; 12).

Although the use of AI in mental health to facilitate clinical decision-making and treatment (13, 14) has shown promise, the VHA has not reported the use of generative artificial intelligence-large language models (GAI-LLMs) in their AI portfolio (7). Generative AI-LLMs build on ML strategies (e.g., Deep Learning and Neural Networks) to construct narrative, human-like responses based on information from a broad range of training sources, including internet web pages, art, and books, and response ratings provided by humans or smaller large language models (LLMs; 8). GAI-LLM applications have been evaluated against mental health professionals (MHCPs) to assess their accuracy in detecting various clinical conditions and making treatment recommendations, with some success noted. In a study that assessed the ability of GAI-LLMs (e.g., ChatGPT-3.5, 4, Bard [now called Gemini]) to recognize major depression as compared to human physicians, the GAI-LLMs were accurate in identifying major depression, less likely to make biased treatment recommendations, and more likely to make treatment recommendations consistent with evidence-based guidelines (15). Other investigations have also found that GAI-LLMs are more accurate than humans in identifying mental disorders, such as Borderline Personality Disorder, and making evidence-based treatment recommendations (16). These findings are intriguing because GAI-LLMs have the potential to identify those at risk for suicide and provide immediate feedback to MHCPs, which is quicker than the risk identification provided by ML approaches. This represents a significant advancement in AI capabilities and utility and is critical as investigations indicate that healthcare professionals are willing to follow recommendations made by GAI-LLMs (17).

In addition to GAI-LLMs’ potential to assess for the presence of mental disorders, a growing body of literature suggests GAI-LLMs have the ability to identify suicide risks and probability of suicide attempts. A study conducted by Levkovich and Elyoseph (18) investigated ChatGPT-3.5 and 4’s ability to assess suicide ideation and risk as compared to MHCPs. Both GAI-LLMs were provided vignettes of a character displaying either high or low perceived burdensomeness and thwarted belongingness – prominent theoretically-based suicide risks (19). Compared to MHCPs, ChatGPT-4 predicted risk for suicide attempts at a similar rate; however, ChatGPT-3.5 underestimated suicide attempt risk. When assessing risk for suicidal ideation, ChatGPT-3.5’s ratings were similar to MHCPs, while ChatGPT-4’s risk ratings were higher. ChatGPT-4 reported that vignette characters experienced more intense emotional pain than MHCPs, while both GAI-LLMs reported lower resilience compared to MHCPs. The findings were attributed to the varying definitions and conceptualizations of emotional pain, resilience, and the nuances in clinical cases that current GAI-LLMs may not capture as effectively as MHCPs. Specifically, it can be speculated that these “soft” risks (e.g., thwarted belongingness) require inferences from contextual elements experienced by individuals, such as stressors and their associated emotional, cognitive, and behavioral responses, which GAI-LLMs may struggle to identify because they are not based on specific, identifiable behaviors. Nonetheless, the findings indicate that GAI-LLMs have potential utility in recognizing suicide risks.

More recently, Shinin-Altman and colleagues (20, 21) built off this work and added several other suicide risks to vignettes to determine if this information would affect GAI-LLMs’ risk assessment for suicide ideation, attempts, or death by suicide. When a history of depression or access to weapons was added to vignettes, Shinin-Altman and associates (21) found that ChatGPT-4 rated characters at an increased risk for suicide ideation, attempts, and death. These interactions were not statistically significant for ChatGPT-3.5; however, ChatGPT-3.5 rated having a history of depression as increasing risk for suicide attempts and death by suicide. Interestingly, ChatGPT-3.5 only rated vignette characters at an increased risk for suicide attempts, but not suicidal ideation or death by suicide, when the characters were described as having access to a weapon. Across all vignettes, ChatGPT-4 rated risks higher than ChatGPT-3.5, but no gender differences were found to be significant. When considering previous suicide attempts, age, or gender as risks, Shinan-Altman and colleagues’ (20) findings were also nuanced. Although age did not seem to affect any risk ratings, several interactions for gender and previous suicide attempts were found to be significant. For ChatGPT-4, the gender by previous suicide attempt interaction was associated with higher estimated risk for suicide attempts for women. ChatGPT-3.5 identified an increased risk for serious suicide attempts and suicidal ideation for men with a history of previous attempts. ChatGPT-3.5 also indicated a higher risk of serious suicide attempt, while ChatGPT-4 rated a higher risk for suicidal ideation for vignettes with a history of previous suicide attempts. In sum, these findings generally demonstrate that the addition of risk factors in vignettes influences GAI-LLMs’ suicide risk assessment; however, there remains variability across GAI-LLMs and room for improvement in GAI-LLMs’ recognition of risks that are inferred from clinical context.

The findings by Shinan-Altman and colleagues (20, 21) highlight the potential utility of GAI-LLMs in assessing suicide risk. However, it remains undetermined whether GAI-LLMs can effectively utilize human-developed models to identify suicide risks and make treatment recommendations. With human MHCPs, evidence demonstrates that structured professional judgment models, which provide a framework for systematically assessing and using suicide risks to make intervention recommendations, are effective strategies for detecting suicide risks and attempts as well as retrospectively predicting death by suicide (22–24).

If GAI-LLMs can leverage an effective suicide risk assessment model, it may enhance the recognition of suicide risks and improve clinical decision-making for MHCPs if integrated into clinical practice. Over the years, various models for assessing suicide risk have been developed, each varying in its focus on patients’ demographic and clinical characteristics (11, 23, 25, 26). Contemporary approaches to suicide risk assessment encourages a multimethod approach combining both actuarial (rating scales) and clinical assessment strategies (clinical interviews), with professional guidelines advocating for the assessment of evidence-based risk and warning signs (11). However, assessing suicide risk poses challenges for MHCPs due to the complexity of balancing risks, protective factors, mental and medical histories, and patients’ preferences for care (5, 27, 28).

To address this complexity and improve suicide risk assessment of veterans, the Veterans Health Administration (VHA) implemented evidence-based, standardized suicide screenings in 2018. This initiative, known as the Suicide Risk Identification Strategy (Risk ID), integrated standardized suicide risk screening with a comprehensive evaluation for veterans who receive a positive screening score from an actuarial measure. Risk ID evolved over several years, and now utilizes the Columbia Suicide Severity Rating Scale screener (C-SSRS; 29), followed by the Comprehensive Suicide Risk Evaluation (CSRE; 30) for veterans scoring in the critical range on the C-SSRS. The CSRE is a semi-structured interview facilitating identification of suicidal risks, a history of suicidal behaviors, and preparatory actions, along with guidelines for treatment that is informed by a risk stratification table that explicitly links identified suicide risks to treatment recommendations. In November 2020, Risk ID was fully implemented across all veterans receiving VHA services, achieving universal assessment with over five million veterans screened for suicide between 2018 and 2019 (31). The implementation of Risk ID was shown to increase veteran contact and engagement with VHA mental health care services (32).

Within Risk ID, the inclusion of a risk stratification table was considered to be an important element for guiding MHCP’s decisions about veterans’ treatment following screening and assessment. The risk stratification table is a key feature of a structured professional judgment model given the explicit linkage between risk assessment and treatment planning. Based on the work of Wortzel and colleagues (28), the risk stratification table emphasizes the use of suicide risk severity and temporality to inform treatment recommendations, balancing care needs with treatment recommendations in order to suggest care in the least restrictive environment. This multidimensional model represents an improvement over previous models, which rate risk solely based on symptom severity, and failed to link risks with treatment need. According to the risk stratification table, acute and chronic risks are associated with varying levels (low, intermediate, and high) that are best addressed with specific treatment options (e.g., inpatient, intensive outpatient, or treatment as usual). Acute suicide risk refers to suicidal ideation that lasts for a short period, typically over minutes to days, in combination with suicide risks and warning signs at various severity levels. High acute risk is associated with suicidal ideation and the inability to remain safe without external support, whereas intermediate acute risk may require psychiatric hospitalization or intensive outpatient treatment depending on the presence of the suicidal intent, identified reasons for living, and/or severity of psychiatric symptoms. In contrast, chronic risk lasts longer, extending beyond days, and include various levels of risks and warning factors associated with suicide. Intermediate chronic risk may involve numerous suicidal risks such as substance misuse, housing instability, and medical conditions occurring in the presence of protective factors (e.g., wanting to live for children and/or religious beliefs). The risk stratification table was formalized by the Veterans Administration’s Rocky Mountain Mental Illness Research, Education, and Clinical Center (MIRECC; 33), which offers extensive training on its implementation across the VHA.

To date, one investigation has assessed the efficacy of the risk stratification table in guiding MHCPs’ identification of acute/chronic risks and treatment planning. In order to do so, Litschi and colleagues (34) first convened a panel of experts to develop six standardized training vignettes representing veterans of diverse ages, racial/ethnic identification, branch of military service, and acute/chronic risks consistent with the risk stratification table and research literature about veteran suicide risks. The vignettes were distributed to clinicians providing services to veterans for evaluation followed by distribution to veteran suicide researchers. Both groups rated the vignettes on acute and chronic risks using the risk stratification table and provided feedback for better alignment. Based on these ratings, consensus was reached for four vignettes, and two were modified further to achieve alignment with the risk stratification table. For the investigation of use of the risk stratification table, the finalized vignettes were distributed to MHCPs employed by the Cohen Veterans Network, which provides outpatient mental health care treatment to military service members, veterans, and their family members. Approximately 42 MHCPs (social workers, counselors, and psychologists) responded to an online survey in which they were randomly assigned to read four of the vignettes and rate the veterans’ acute and chronic risks using the risk stratification table. Additionally, they identified treatment plans based on risk ratings. Litschi and associates (34) found that the acute and chronic risk ratings made by MHCPs mostly aligned with the pre-investigation clinicians’ and researchers’ ratings. Participants’ treatment decisions varied in relationship to vignette characteristics, but not the MHCPs’ background (e.g., mental health profession or familiarity with the risk stratification table), indicating that MHCPs from multiple disciplines and training backgrounds could apply the risk stratification table for making treatment decisions. Moreover, Litschi et al. (34) found that acute risk perception was associated with treatment disposition; vignettes with the highest acute risk ratings were more likely to be recommended for hospitalization, partial hospitalization, or intensive outpatient treatment. MHCPs reported in qualitative responses that 1) the perception of chronic risk influenced acute risk identification, 2) a variety of factors were used to determine treatment disposition other than acute risk perception, and 3) MHCPs varied in their understanding and application of critical concepts such as suicide intent and suicide preparatory behaviors. Litschi and colleagues’ investigation demonstrated that standardized vignettes may be useful in assessing clinical decisions, such as treatment planning, and the risk stratification table facilitated assessment of veteran suicide risk.

The findings from this investigation are intriguing because they demonstrate that MHCPs can benefit from the use of a stratified risk assessment model to assign risk ratings and plan treatment commensurate with those risks. What has yet to be assessed is whether GAI-LLMs can apply a risk assessment model to accurately detect suicide risk and make appropriate treatment recommendations similar to MHCPs. With GAI-LLMs demonstrating promise that they can facilitate clinical decisions in detection of mental disorders [Major Depressive Disorder (15) and Borderline Personality Disorder (16)] and suicide risks (18, 20, 21), exploring the ability of GAI-LLMs to apply a risk stratification model is critical as GAI-LLMs are being increasingly incorporated into mental health practice (13). It is also important to assess GAI-LLMs’ ability to follow human assessment models as research has found that healthcare providers rely on risk assessments made by GAI-LLMs, even if these assessments are biased (17). Given this, our investigation used an innovative approach combining GAI-LLM suicide risk assessment with the use of standardized vignettes of veterans and the MIRECC’s risk stratification table (33) to determine if GAI-LLMs are able to identify suicidal risk and make treatment recommendations comparable to MHCPs. Based on the previous findings, we expected that 1) GAI-LLMs’ acute and chronic risk ratings would be similar to MHCPs, 2) GAI-LLMs would make more restrictive treatment recommendations for standardized vignette characters with higher acute and chronic risks, and 3) GAI-LLMs’ acute and chronic risk ratings would predict treatment disposition decisions. We also assessed differences in acute and chronic risk assessment across GAI-LLMs, but made no specific predictions as the previous research has shown that GAI-LLMs’ risk assessments vary widely across investigations, which are likely due to variations in GAI-LLM training.

## Methods

### GAI-LLMs and human participants

The GAI-LLMs selected for this investigation included ChatGPT-3.5, ChatGPT-4o, and Google Gemini. These GAI-LLMs were selected based on their name recognition, frequency of use, accessibility, and high ratings in reasoning, language, and instruction following assessments (35, 36).

All responses provided by the GAI-LLMs were compared to MHCPs’ ratings (N = 42; 34) of the same vignettes. The MHCPs’ responses were taken from Litschi and colleagues’ (34) investigation. In this investigation, Litschi and colleagues recruited participants from a network of clinics across the United States providing mental health care services to active duty service members, veterans, and their family members. A total of 161 MHCPs were invited to participate in the online investigation, and a total of 42 completed the investigation. Participants did not receive compensation for participation. A majority of the MHCPs identified as women (88.1%) and were social workers (43.9%), marriage and family counselors (19%), professional counselors (38%), and psychologists (4.7%). Approximately 90% reported having two or more years of mental health care experience and most had some familiarity with the risk stratification table (73.8%). Approximately 30.9% reported at least one previous client surviving a suicide attempt during services or after termination, and 11.9% experienced a client dying by suicide during services or after termination. Because the GAI-LLMs’ responses were compared to published data, there was no need for informed consent for this investigation.

### Materials

Four standardized vignettes about fictional veterans (Bill, Darrell, Lupe, and Linda; 34, 37) were used to assess the GAI-LLMs’ identification of suicide risks. The vignettes reflected veterans with a range of acute and chronic risks, racial/ethnic identification (White, Black, and Latine), age (28-55 years of age), gender (two men and two women), and military service branch (United States Marine Corps, Army, Coast Guard, and Air Force). As an example of vignette content, Bill, the most acute and chronic risk vignette, was described as a 55-year old white man, who had experienced a relationship break-up and was homeless. He was also described as having several previous psychiatric hospitalizations, suicide attempts, and current suicidal ideation. Bill had immediate access to guns, rehearsed shooting himself, and refused to discuss securing his weapons. All vignettes used in the investigation are provided online (37). From previous research (34), MHCPs rated the veterans’ risks as follows: 1) Bill: high acute and chronic risk; 2) Darrell: high acute risk, intermediate chronic risk; 3) Lupe: intermediate acute risk, high chronic risk; 4) Linda: high acute risk, low chronic risk.

### Procedures

A zero-prompt approach was used, meaning the GAI-LLMs were given prompts to respond to without example responses or iterative training. This approach is an effective strategy in revealing GAI-LLM capabilities because the output is reflective of its neutral performance (38). For each trial, the GAI-LLM was opened in a privacy browser, provided with a vignette, the risk stratification table, and asked to rate the veteran’s acute and chronic risks. The GAI-LLM was also asked to specify the level of care needed by the veteran. Specifically, all GAI-LLM were 1) directed to read the vignette, 2) review instructions and examples from the risk stratification table for acute risk, and 3) assign the level of acute risk using the rating scale provided (see below) in one prompt. In a second prompt, the GAI-LLM were directed to 1) review instructions and examples from the risk stratification table for chronic risk, 2) assign level of chronic risk, and 3) select treatment disposition for the veteran using the treatment disposition rating scale (see below). All prompts and prompting materials are available from the corresponding author upon reasonable request.

After the GAI-LLM responded, the data was copied and the tab was closed. These steps were repeated ten times with each of the three GAI-LLM with the veterans assessed in a sequential order, ensuring that data collection for each veteran was completed before proceeding to the next. A total of 30 trials (*N* = 120) were generated for each vignette. All data was collected from July 23-July 24, 2024.

### Measures

Acute and Chronic Suicide Risks (34, 37). The GAI-LLMs were asked to rate each veterans’ level of acute and chronic risk (“Using the criteria above, please evaluate this veteran’s ACUTE/CHRONIC risk for suicide”) using a 1 (*Low*) to 9 (*High*) response scale. Using these items, Litschi and colleagues (34) found that higher acute/chronic risk was associated with MHCPs recommending more intensive care needs.

Treatment Disposition (34, 37). The GAI-LLMs were asked to indicate the level of care needed by each veteran (“What is your disposition determination for this veteran?”) using a 1 (*Plan for hospitalization (voluntary/involuntary)*) to 4 (*No further action required, follow-up as usual*) response scale. Litschi and colleagues (34) found that veterans rated with higher acute risk were also rated as needing more intensive treatment.

### Data analysis plan

Acute and chronic risk ratings for each vignette were compared to ratings made by the MHCPs using independent groups *t*-tests. A one-way ANOVA with four levels (severity of veterans’ risks, with Bill the highest and Linda, the lowest) collapsing across GAI-LLMs was used to assess GAI-LLMs’ treatment disposition decisions for the veterans.

For comparison across GAI-LLMs, three one-way ANOVAs with three levels (for GAI-LLM) were used to assess differences in acute risk ratings, chronic risk ratings, and treatment disposition. All statistically significant main and interaction effects from the ANOVAs were assessed using Tukey’s HSD to control for Type 1 error. Hierarchical regression analysis controlling for veterans’ risk (coded 1 - 4; Bill = 1, Darrell = 2, Lupe = 3, and Linda = 4) and GAI-LLM (coded 1 – 3; ChatGPT-3.5 = 1; ChatGPT - 4.o = 2; Google Gemini = 3) was used to assess prediction of treatment disposition by GAI-LLMs’ acute and chronic risk ratings. On the first step, we included veterans and GAI-LLMs as our analyses found differences across these variables. On the next step, acute and chronic risks were added to the model to assess if these variables were predictive of treatment disposition decisions by the GAI-LLMs. All data were screened for outliers and none were detected. All data analyses were completed using JASP version 0.19.2 (39).

## Results

**Figure 1:**
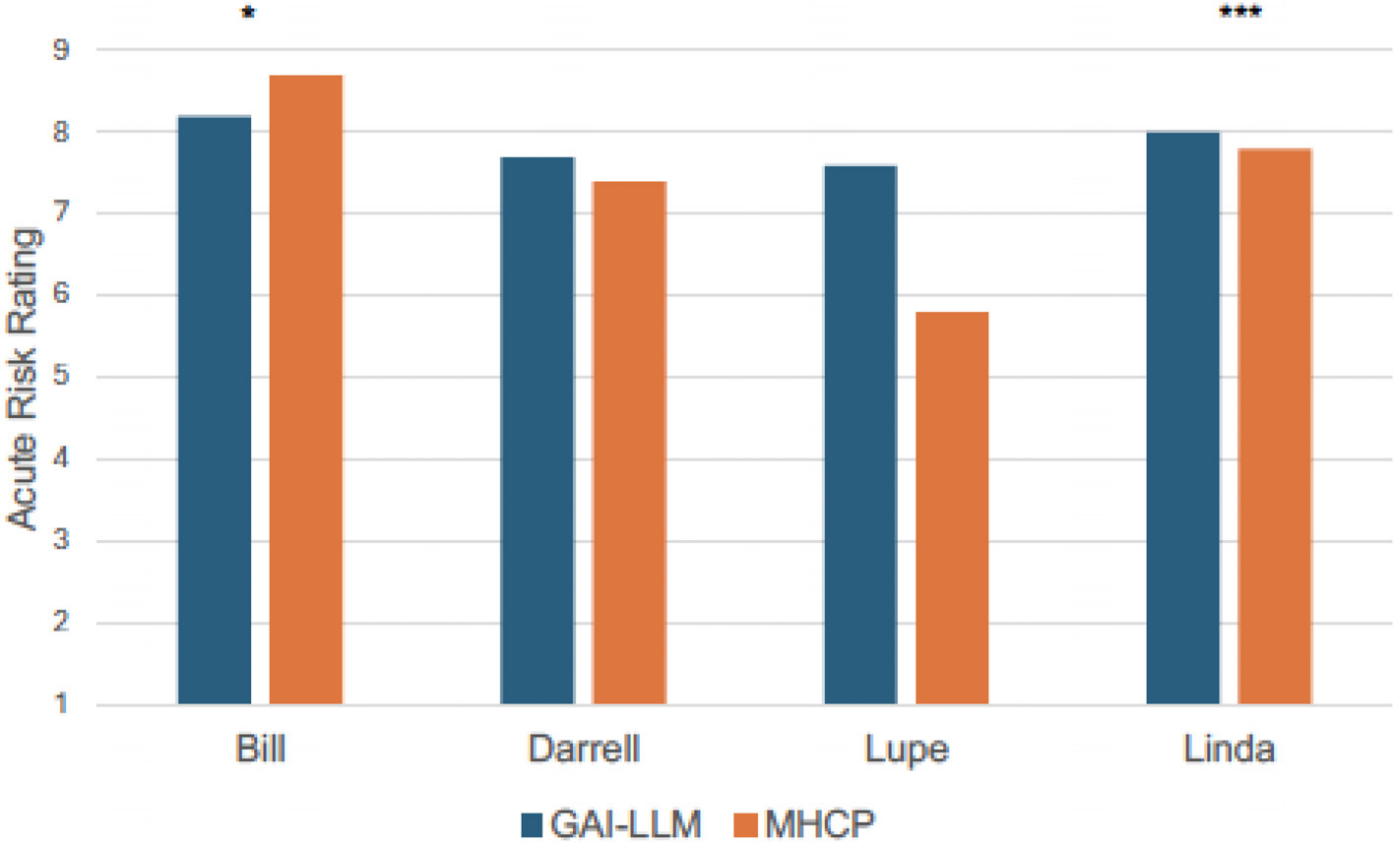
Acute risk ratings by GAI-LLM and MHPs. GAI-LLM, Generative Artificial Intelligence-Large Language Model; MHCPs, Mental health care providers. Total number of GAI-LLM veteran ratings was *n* = 30 per veteran. Total number of MHCPs ranged from *n* = 21 - 32 per veteran. *p <.05. ***p <.001.

**Figure 2:**
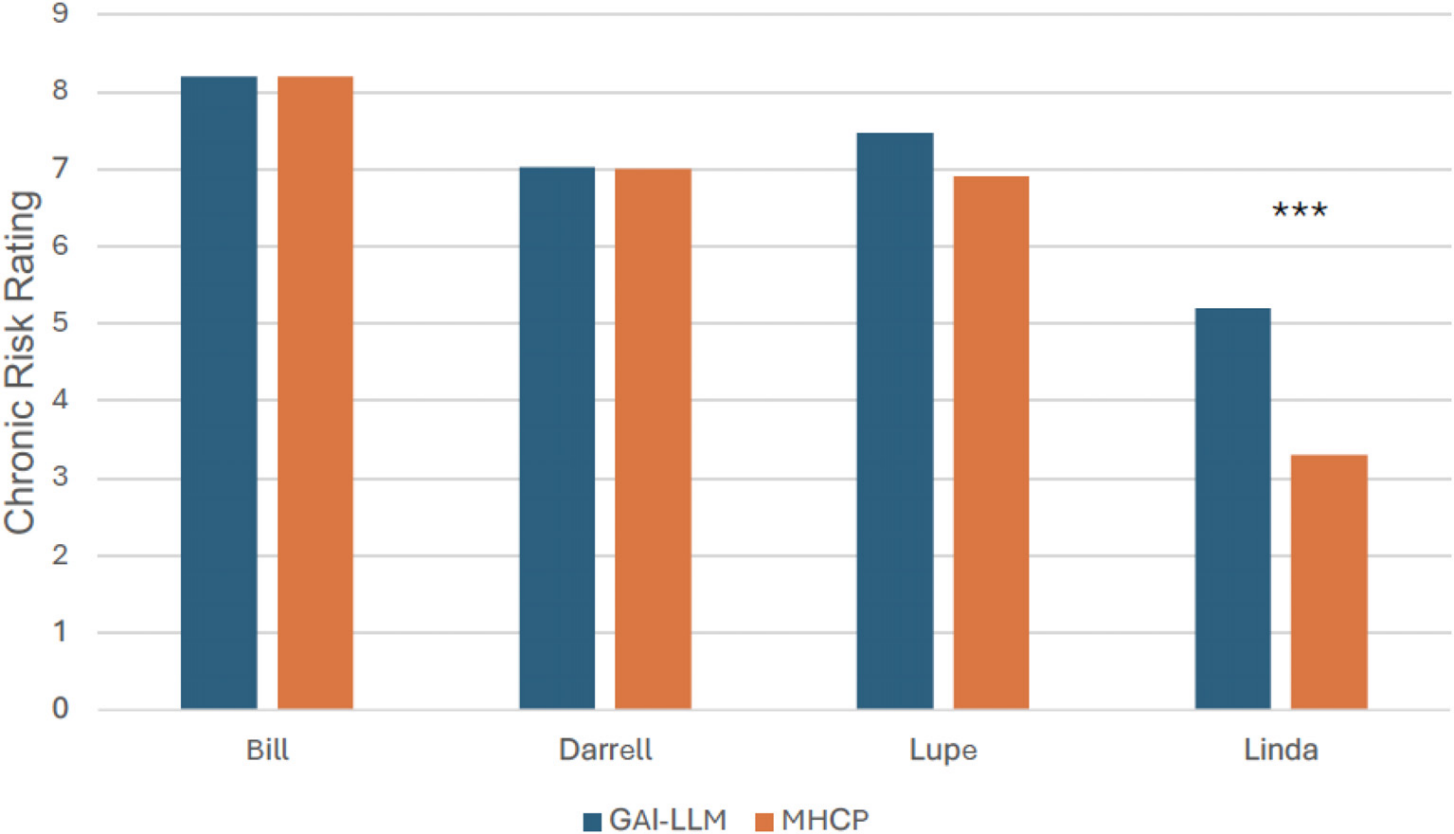
Chronic risk ratings by GAI-LLM and MHPs. GAI-LLM, Generative Artificial Intelligence-Large Language Model; MHCPs, Mental health care providers. Total number of GAI-LLM veteran ratings was *n* = 30 per veteran. Total number of MHCPs ranged from *n* = 21 - 32 per veteran. ***p <.001.

Comparisons of acute risk ratings between GAI-LLMs and MHCPs are shown in **Figure 1**. As seen in **Figure 1**, the most acute vignette rated by MHCPs (Bill; *M* = 8.70, *SD* = 0.60) was rated as less acute by GAI-LLMs (*M* = 8.20, *SD* = 0.96; *t*(60) = 2.48, *p* <.05; Hedge’s *g* = 0.63, 95% CI: 0.12., 1.13). The least acute risk vignette rated by MHCPs (Lupe; *M* = 5.80, *SD* = 1.70) was rated as more acute by GAI-LLMs (*M* = 7.60, *SD* = 0.86; *t*(59) = 5.19, *p* <.001; Hedge’s *g* = 1.31, 95% CI: 0.76., 1.86). There were no other differences between MHCPs and GAI-LLMs in acute risk ratings for the other veterans (all *p*s >.05). Comparisons of chronic risk ratings between GAI-LLMs and MHCPs are shown in **Figure 2**.There were no differences between GAI-LLMs and the MHCPs for most chronic risk ratings (all *p*’s >.05); however, the vignette rated with less chronic risk by MHCPs (Linda; *M* = 3.30, *SD* = 1.50) was rated with more chronic risk by GAI-LLMs (*M* = 5.20, *SD* = 1.83; *t*(49) = 4.35, *p* <.001; Hedge’s *g* = 1.12, 95% CI: 0.58., 1.66).

A one-way ANOVA with four levels of veterans assessing mean differences in treatment disposition collapsing across GAI-LLMs was statistically significant (*F*(3,116) = 12.72, *p* <.001, *η* ^2^ = .25). As seen in **Table 1**, the GAI-LLMs indicated that the veterans with the higher chronic suicide risk (Bill, Darrell, and Lupe) required more intensive treatment than the veteran with the lowest chronic risk (Linda; all *p*s <.01). No other differences between vignettes were found.

**Table 1:** Differences in treatment disposition ratings collapsed across GAI-LLM.

	Standardized Vignettes			
	Bill Mean (SD)	Darrell Mean (SD)	Lupe Mean (SD)	Linda Mean (SD)
Treatment Disposition	1.00(0.00)^a^	1.37(0.56)^b^	1.10(0.31)^c^	1.87(1.01)^a,b,c^

*N*, 120. GAI-LLM, Generative artificial intelligence. Cells with the same superscripts are statistically different.

To assess variation in ratings across GAI-LLMs, three one-way ANOVAs with three levels (GAI-LLM) were calculated for acute risk, chronic risk, and treatment disposition. Means differences across GAI-LLMs are shown in **Table 2**. For acute risk, the overall model was statistically significant (*F*(2,117) = 64.44, *p* <.001, *η* ^2^ = .52). ChatGPT-3.5 rated veterans with less acute risk than ChatGPT-4o or Google Gemini (*p’s* <.001). ChatGPT-4o rated veterans as having more acute risk than Google Gemini (*p* <.001).

**Table 2:** Differences in GAI-LLM for acute risk, chronic risk, and treatment disposition.

	ChatGPT-3.5 Mean (SD)	ChatGPT-4o Mean (SD)	Google Gemini Mean (SD)
Acute Risk	7.05(0.22)^a^	8.73(0.64)^a,b^	7.85(0.92)^a,b^
Chronic Risk	6.80(1.16)	7.58(1.66)^b^	6.55(1.95)^b^
Treatment Disposition	1.25(0.71)^a^	1.00(0.00)^b^	1.75(0.78)^a,b^

GAI-LLM, Generative artificial intelligence large language models. Cells with the same superscript are statistically different from each other.

For chronic risk, the overall model was statistically significant (*F*(2,117) = 4.34, *p* <.05, *η* ^2^ = .07). ChatGPT-4o identified more chronic risk for veterans than Google Gemini (*p* <.05).

A one-way ANOVA assessing treatment disposition was also statistically significant (*F*(2,117) = 15.87, *p* <.001, *η* ^2^ = .21). ChatGPT-4o indicated every veteran should be hospitalized compared to Google Gemini (*p* <.001). ChatGPT-3.5 was also likely to recommend more restrictive treatment than Google Gemini (*p* <.01). The ChatGPT GAI-LLMs did not differ from each other (*p* >.05).

To assess risks associated with treatment disposition decisions by GAI-LLMs, we used hierarchical regression analysis. Prior to the regression analysis, bivariate correlations were calculated to assess intercorrelations between model variables. Acute risk ratings were positively correlated with chronic risk ratings (*r*(128) = .24, *p* <.01). Acute risk ratings were not correlated with treatment disposition ratings (*r*(128) = -.14, *p* >.05), while chronic risk ratings were (*r*(128) = -.64, *p* <.001). On the first step of the hierarchical regression model, we included both veterans and GAI-LLMs. On the second step, we included both acute and chronic risks ratings identified by GAI-LLM. The results are shown in **Table 3** and suggest that chronic risk rating, but not acute, were predictive of GAI-LLMs’ treatment disposition decisions for veterans after controlling for veterans’ level of risks. GAI-LLMs also contributed to the prediction of treatment disposition in the final model and the results suggest that Google Gemini was more likely to recommend less restrictive treatment for the veterans.

**Table 3:** Prediction of GAI-LLM treatment disposition by acute and chronic risks after controlling for veterans and GAI-LLM.

Variable	*β*	*R^2^adj*	*ΔR^2^*
Step 1		.24***	.24***
Veteran	.38***		
GAI-LLM	.30***		
Step 2		.47***	.25***
Veteran	.05		
GAI-LLM	.31***		
Acute Risk	-.11		
Chronic Risk	-.56***		

GAI-LLM, Generative artificial intelligent programs. Veteran coded 1 - 4; Bill, 1, Darrell, 2, Lupe, 3, and Linda, 4. GAI-LLM coded 1 – 3; ChatGPT-3.5, 1; ChatGPT-4o, 2; Google Gemini, 3.

***p <.001.

## Discussion

This investigation aimed to evaluate the capabilities of various GAI-LLMs, specifically ChatGPT-3.5, ChatGPT-4o, and Google Gemini, in using the VHA’s risk stratification table to identify acute and chronic suicide risks and propose treatment plans aligned with the identified risks. We also assessed if GAI-LLMs’ suicide risk assessments matched those of human MHCPs who provide mental health services to veterans. Our investigation represents an important innovation by incorporating standardized vignettes to compare GAI-LLMs’ suicide risk assessment performance to that of MHCPs. This methodological approach enhances the evaluation of GAI-LLM capabilities through accurate assessment against established standards. Prior research has not utilized this approach, resulting in a significant gap in understanding GAI-LLMs’ ability to identify suicide risks. This is crucial as recent data indicates that suicide rates for veterans in the United States are disproportionately higher compared to the general population, and early detection and prevention of suicidal risks and behaviors can be critical to reducing suicide deaths (3, 40). Although GAI-LLM use in the mental health field is relatively novel, interest from healthcare providers (41) and the public (42, 43) is growing rapidly, necessitating a better understanding of the capabilities and limitations of GAI-LLMs in mental health care. Given the increased integration of AI into mental health care and suicide risk detection, this study provides a necessary examination of GAI-LLMs’ efficacy in acute and chronic suicide risk assessments and treatment recommendations.

Based on previous literature (18), it was expected that GAI-LLMs would assess acute and chronic suicide risk similarly to MHCPs when using the VHA’s risk stratification table, which is a critical element of a structured professional judgment model for suicide prevention. This hypothesis was mostly supported as our investigation found that GAI-LLMs’ ratings only slightly differed from those of MHCPs. For acute risk ratings, the GAI-LLMs differed on two of the vignettes compared to MHCPs. For the standardized vignette rated as most acute by MHCPs, GAI-LLMs’ ratings were slightly less acute; however, GAI-LLMs still rated this vignette as having the highest acute risk compared to the other vignettes. GAI-LLMs also differed significantly in the acute risk rating for the vignette rated as least acute by MHCPs. Here, GAI-LLMs rated this veteran’s acuity as greater than the MHCPs, but the GAI-LLMs’ ratings were lower compared to the other vignettes, which was consistent with the pattern of ratings made by MHCPs (34). When comparing GAI-LLMs’ chronic risk ratings to those of MHCPs, the GAI-LLMs only differed on one vignette, which was rated as having higher chronic risk than ratings made by the MHCPs. These findings are largely consistent with recent investigations demonstrating that GAI-LLMs perform similarly to MHCPs in recognizing mental health disorders (15, 16) but shows greater variation in identifying suicide risks (18). These results suggest that GAI-LLMs are likely more accurate at tasks involving recognition of discrete symptoms mapping onto specific criteria, but may suffer performance decrements when attempting to identify variables, such as risks, that require inferences from clinical context. That is, “soft” risk factors may pose a bit more of a challenge for GAI-LLMs to accurately identify. This is not likely due to GAI-LLMs having difficulty with understanding and predicting human emotion as past investigations indicate that GAI-LLMs are adept at such tasks (44), but rather it may be that integrating contextual elements (e.g., stressors, thoughts, and emotions) to reliably recognize suicide risk may be at the upper limit of GAI-LLMs’ inferencing abilities. Notably, MHCPs also report difficulty in making decisions when “soft” risk factors are involved and vary in the weight they assign to risk and protective factors, even when using structured or standardized assessments and materials (34).

Two critical implications from these findings are GAI-LLMs’ need for continued human MHCPs’ oversight to ensure the relevance and accuracy of assessments. Also clear from our results is that GAI-LLMs require on-going training to improve performance in detection of risk factors for psychopathological conditions. It is comforting to see that GAI-LLMs tended to make mostly more conservative risk ratings than MHCPs, although such ratings could lead to inappropriately restrictive mental health care. Numerous GAI-LLMs applications have demonstrated that effective training for processes in mental health care are possible (e.g., providing reflections; 13), suggesting that GAI-LLMs’ suicide risk identification can be improved. With continued advancement in GAI-LLMs’ capabilities, especially with processing human spoken language, it is easy to conceive that GAI-LLMs may be integrated into professional service appointments, such as assessments and psychotherapy, as a real-time “co-pilot” that is able to synthesize session content and flag noteworthy thoughts, behaviors, and emotions relevant for clinical consideration, including those critical for suicide risk. Proprietary applications using GAI-LLMs have emerged that are able to summarize and perform sentiment analysis of narrative content provided by clients between psychotherapy sessions for use in psychotherapy sessions. It is easily conceivable that MHCPs could use this technology in real-time to identify any risks that may need further evaluation or intervention, providing an improvement over ML strategies which take much longer to provide critical information.

Although GAI-LLMs are in need of continued training and have certain limitations requiring human MHCP oversight, it is also important to address the practical implications of implementing GAI-LLMs as a support tool in practice. In particular, MHCPs may benefit from receiving training in how to use and prompt GAI-LLMs effectively. They also will benefit from developing familiarity with GAI-LLMs’ capabilities and limitations, and using caution when interpreting GAI-LLMs’ outputs as bias and stigma have been detected in their narratives. These capabilities fall under the broad umbrella of GAI-LLM literacy, which has been investigated and discussed in primary and secondary education for years, but extension of these concepts to adults, and MHCPs, has been limited.

Specifically, MHCPs need to develop the GAI-LLM literacy competencies of awareness, usage, evaluation, and ethical understanding through education and training (45). To facilitate awareness, MHCP’s must learn how GAI-LLMs have been integrated into numerous applications, particularly those used to provide mental health care and support to people in need of mental health services (13). Usage education would entail providing descriptions of GAI-LLM specific applications for mental health, including the development of GAI-LLM applications to assess for anxiety and depression as well as GAI-LLM that provide psychotherapy (13). Critical to this discussion is the strength and limitations of the evidence-base for GAI-LLMs providing mental health assessment and treatment. Evaluation entails encouraging MHCPs to recognize that GAI-LLM generated information may seem compelling, but has substantial limitations given that it can be inaccurate, show bias, and express public stigma. MHCPs using GAI-LLMs should be encouraged to verify any information received from GAI-LLMs with credible sources. Finally, MHCPs using GAI-LLMs should be informed about the ethical concerns of GAI-LLM use. Of these, knowledge of how user’s data, including questions posed to GAI-LLMs and users’ responses to GAI-LLMs, are used to develop and train GAI-LLMs is critical as confidentiality of user’s data is not guaranteed (13). Potential MHCP GAI-LLM users need to know this information so they can make informed decisions about whether they should incorporate GAI-LLMs into professional service activities in light of ethical and legal standards. At a minimum, MHCPs who want to use GAI-LLMs need to search for those applications that meet legal requirements for electronic confidentiality.

Our investigation also assessed GAI-LLMs’ ability to select appropriate treatment recommendations given the risks identified from the vignettes, which has not been attempted in previous GAI-LLM investigations (20). Our results were mostly consistent with our hypotheses and demonstrated that GAI-LLMs tended to select more restrictive treatment plans for veterans when chronic risk was elevated. Interestingly, these findings dovetail with Litschi and colleagues (34) suggesting that MHCPs’ treatment disposition ratings were associated with acute risk ratings, which were strongly influenced by chronic risk ratings. The implication is that GAI-LLMs, like humans, may emphasize chronic risk assessment in determining appropriateness of treatment. The reasons for these findings are unclear. Again, on-going (16) and past research (15) suggest that GAI-LLMs are adept at selecting evidence-based interventions for a variety of mental health conditions. It is possible that the GAI-LLMs in this investigation demonstrated a preference for addressing long-term risks as a means of treatment planning for suicide, which suggests that GAI-LLMs may need further training to incorporate acute risks in developing treatment recommendations to provide support in the least restrictive environment. It is also possible that the order in which GAI-LLMs were asked to make decisions about acute and chronic risks may have influenced treatment dispositions decisions. In our investigation, the chronic risk assessment was the decision GAI-LLMs made just prior to providing treatment disposition, as it was in the original Litschi and colleagues’ (34) investigation with MHCPs. Thus, GAI-LLMs may have used its chronic risk ratings as a guide for treatment planning. The VHA risk stratification table explicitly informed GAI-LLMs that if a vignette showed high acuity, then inpatient hospitalization would likely be necessary, bringing into question what led GAI-LLMs to prioritize chronic risk over acute risks ratings in determining treatment disposition. Regardless, the data emphasizes concerns regarding GAI-LLMs’ ability to accurately assess risks and use them to make informed treatment decisions.

The present investigation also revealed variations in GAI-LLMs’ performance, which is consistent with past research (18, 20, 21). ChatGPT-3.5 had lower ratings for acute risk compared to ChatGPT-4o and Google Gemini. However, ChatGPT-4o rated veterans as having more acute and chronic risks compared to Google Gemini and recommended hospitalization for every veteran. This treatment disposition was seen as more restrictive compared to the other two GAI-LLMs, which, on average, recommended outpatient treatment. These findings reflect that there are important variations between GAI-LLMs, which are likely due to differences in training for each model. GAI-LLM training is considered proprietary and guarded as trade secrets in GAI-LLM development, which limits appreciation of how GAI-LLM training may affect its responses. If GAI-LLMs use are continued in mental health applications, which in all likelihood it will be, GAI-LLM developers are strongly encouraged to incorporate MHCPs and individuals with lived experiences (e.g., people who have experienced mental health disorders, people who have experienced suicide risks, and veterans) into their GAI-LLM training processes in order to address how GAI-LLM prioritizes information when making decisions about mental health risks and treatment.

The present investigation has several limitations to be considered when considering the findings. One such limitation was the inability to counterbalance important factors, such as gender, race/ethnic identification, and age, presented in the vignettes. This leads to potential confounds when considering the results given that these factors were inseparable from the acute/chronic risks ratings. Future investigations should assess how these factors may shape GAI-LLMs’ responses given that other investigations have found that GAI-LLMs will show biased responses when provided these characteristics (16, 20, 21, 46, 47). These findings are not surprising as GAI-LLMs training sources are human derived, meaning that biases and stigma are incorporated into GAI-LLM. It is hoped that GAI-LLM developers will work to incorporate mental health care professionals and people with lived experiences into their development of GAI-LLM applications used for mental health as a means of identifying and reducing the presence of bias and stereotypes incorporated into GAI-LLMs’ algorithms. Related to this, GAI-LLM developers are encouraged to provide greater transparency about their training sources and to incorporate strategic prompting strategies to allow for the systematic identification of bias and stereotypes so these can be addressed in training. Methodical use of red-teaming (48) and incorporation of multiple GAI-LLM guardrails (in which GAI-LLM’s responses are evaluated multiple times prior to release) would also be useful in reducing biased GAI-LLM responding (49). Consideration of these approaches are critical given that many in the general population value GAI-LLMs’ responses (50) and people with stigmatized conditions prefer GAI-LLM interventions to avoid stigmatization by human MHCPs (42, 43). This suggests that bias and stereotypes expressed by GAI-LLMs has the potential to result in substantial harm to unsuspecting users who are turning to GAI-LLM for help.

An additional noteworthy limitation is that when working with GAI-LLMs, research findings are time limited given that GAI-LLM are continuously evolving and developers frequently release updated applications. Newer versions of GAI-LLMs do not always mean improvement, although that tends to be the trend (51). To address this limitation, future investigators are encouraged to replicate and extend our results with newer versions of the GAI-LLM used in this investigation. Another avenue of future research would be to iteratively assess GAI-LLMs’ training to determine if risk assessment and treatment recommendations can be improved. Effective use of GAI-LLMs is highly dependent on the prompts used, and a variety of iterative prompt strategies could be used to fine-tune GAI-LLMs’ performance in identifying suicide risks and treatment planning (38). Future efforts can also look into developing strategies for GAI-LLMs to enhance its ability to effectively weigh risk factors when determining treatment disposition. As part of this process, careful attention should be paid to the information that GAI-LLMs are using to assign acute and chronic risks to determine if these are consistent with the way risks have been conceptualized in the suicide prevention literature. With the increased integration of GAI-LLMs into the mental health field and the potential for use of GAI-LLMs to provide real-time risk identification and psychotherapy, additional training is needed to capitalize on the promise GAI-LLMs offer.

A final limitation of our research was the reliance on standardized vignettes. These vignettes contained information designed to assist in making acute and chronic risk assessments, aligning with our aim to evaluate whether GAI-LLMs could perform comparably to MHCPs. However, we did not evaluate GAI-LLMs’ performance using real-world clinic information, where crucial information about acute and chronic risks might be missing. This limitation affects the generalizability of our findings and we recommend future studies to explore GAI-LLMs’ effectiveness using clinical data from real-world environments to better assess its potential generalizability to clinical practice.

Overall, the current investigation demonstrated that when given a suicide risk assessment model to follow, GAI-LLMs are able to identify acute and chronic suicide risks in veterans similarly to MHCPs, with some variation. This reflects the potential of GAI-LLMs to be further incorporated into mental health practice while highlighting the need for on-going training to fine-tune GAI-LLM performance. It is important to note that GAI-LLMs show a gap in assessing what factors should determine treatment disposition, emphasizing its current limitations in evaluating the next steps when presented with veterans with suicidal ideation. All in all, these findings contribute to the ever-growing body of GAI-LLM literature and, most importantly, the results suggest that GAI-LLMs have the promise of providing real-time identification of suicide risks and treatment recommendations. However, further refinement and on-going human MHCP oversight is critical.

## Data Availability

The raw data supporting the conclusions of this article will be made available by the authors, without undue reservation.
